# “PROUD to have been involved”: an evaluation of participant and community involvement in the PROUD HIV prevention trial

**DOI:** 10.1186/s40900-020-00189-3

**Published:** 2020-04-17

**Authors:** Mitzy Gafos, Annabelle South, Bec Hanley, Elizabeth Brodnicki, Matthew Hodson, Sheena McCormack, T. Charles Witzel, Justin Harbottle, Claire Vale

**Affiliations:** 1 Department of Global Health and Development, London School of Hygiene and Tropical Medicine, London, WC1H 9SN UK; 2 MRC Clinical Trials Unit at UCL, 90 High Holborn, London, WC1V 6LJ UK; 3 NAM / aidsmap, Unit 19, St Mark’s Studios, 14 Chillingworth Road, London, N7 8QJ UK; 4 Sigma Research, Department of Public Health, Environments and Society, London School of Hygiene & Tropical Medicine, London, UK; 5 SH:24 CIC, 35a Westminster Bridge Road, London, SE1 7JB UK

**Keywords:** PPI, Participant involvement, Pre-exposure prophylaxis, “Patient and public involvement”

## Abstract

**Background:**

The PROUD trial, a HIV prevention trial in men who have sex with men and trans women, set out to involve community representatives and trial participants in several ways. PROUD also aimed to evaluate participant involvement, to learn lessons and make recommendations for future clinical trials.

**Methods:**

Two structured surveys, one of participant and community representatives involved in the PROUD study, and the other of researchers from the PROUD team, were carried out in 2017. The results from the surveys were reviewed quantitatively and qualitatively, and themes emerging from the data identified and synthesised.

**Results:**

Survey invitations were sent to 88 involved participants, 11 community representatives and 10 researchers. The overall response rate was 55% (60/109). Overall, participants were younger than community representatives, and the majority were from Greater London. As expected, participants were predominantly involved in participant involvement meetings and community representatives in management committees.

Participants and community representatives cited different motivations for getting involved in PROUD. Overall, participants were positive about their involvement; only two participants rated their experience unfavourably. Community representatives were also broadly positive. Most participants and all community representatives felt their involvement made a difference to the trial, themselves and / or the organisations they represented. However, some participant answers reflected the impact of participation in the trial rather than involvement in PPI activities.

Researchers felt that PPI had positive impact across the entire trial cycle. Half felt they would have liked there to have been more PPI activity in PROUD. Researchers noted some challenges and recommendations for the future, including need for adequate funding, more engagement in PPI by all researchers, the need for PPI expertise to facilitate involvement activities and training and mentoring in PPI.

**Conclusions:**

Involving clinical trial participants and wider community representatives as active partners in PPI is feasible and valuable in trials. Researchers are encouraged to consider and appropriately resource participant involvement and prospectively evaluate all PPI within their trials.

## Plain English summary

The PROUD trial tested how good pre-exposure prophylaxis (PrEP) was at preventing HIV in men who have sex with men and trans women. Pre-exposure prophylaxis is the use of antiretroviral drugs by HIV-negative individuals to reduce the risk of acquiring HIV. Community representatives were involved in the design of the trial and the plan to involve trial participants as active partners. PROUD also aimed to review the impact of community and participant involvement on the trial, and to share lessons for future studies.

Involved participants, community representatives and researchers evaluated their involvement by completing surveys. There was a difference between what motivated community representatives and participants to get involved in PROUD and between how the two groups found the experience. Community representatives were very positive, as were nearly all participants. More than three quarters agreed there were benefits of involvement. Researchers were also very positive about the involvement and the impact it had on PROUD. Some felt that more patient and public involvement (PPI) in PROUD would have been better. In addition, they noted some areas for improvement in future, including better resourcing and more support for PPI activities.

Involving trial participants in PPI is unusual in the United Kingdom. Participant meetings were held during PROUD, as well as representation on study oversight groups, and our evaluation demonstrated the benefits of this. We encourage others to consider it in future clinical trials.

## Background

There is a long tradition of stakeholder involvement in Human Immunodeficiency Virus (HIV) treatment research [1, 2]. However, in the early part of the 2000’s, the failure to start, and premature closure, of two HIV prevention trials evaluating oral pre-exposure prophylaxis (PrEP) in Nigeria, Cameroon and Cambodia was attributed in part to a lack of community-level stakeholder involvement [3]. This led to a series of Joint United Nations Programme on HIV/AIDS (UNAIDS) consultations, which culminated in the publication of ‘good participatory practice’ (GPP) guidelines [2]. These guidelines mandated the involvement of a broad range of stakeholders, including trial participants, throughout the life-cycle of HIV prevention trials.

The PROUD (PRe-exposure Option for reducing HIV in the UK: an open-label randomisation to immediate or Deferred daily Truvada for HIV negative gay men) HIV prevention trial was a pragmatic open label randomised controlled trial evaluating the benefit of pre-exposure prophylaxis (PrEP) in the prevention of HIV for men who have sex with men (MSM) and trans women [4–6]. Although PrEP was licensed in 2012 in the USA, the UK sexual health professional bodies called for additional evidence on the benefit of PrEP before recommending its inclusion in clinical guidelines [7]. PROUD was conducted in accordance with GPP guidelines. For the purpose of this study, we use the INVOLVE definition of patient and public involvement (PPI): “Research being carried out ‘with’ or ‘by’ members of the public rather than ‘to’, ‘about’ or ‘for them’” [8]. A range of PPI models were implemented throughout the design, implementation, analysis and dissemination phases of PROUD, which have been previously reported [9, 10] and are summarised in Table 1. Community representatives from regionally diverse HIV and gay men’s sexual health charities were invited to join an e-group along with researchers, clinicians, commissioners and policy makers, to design a trial that was both acceptable to potential participants and able to address the relevant research questions and ultimately to influence policy and practice. Community representatives remained involved in trial management and oversight committees throughout the life-course of the trial. These groups met through face-to-face meetings and teleconferences. PPI activities were coordinated by the trial social scientist.

**Table 1:** PPI activities in PROUD

	PPI Activity	Area of impact (based on PiiAF [11])
**1.**	Membership of e-group to design trial (> 10 community members)	• Agenda • Research design
2.	Membership of advisory committees: • Community engagement group (CEG) (involved in development of study documentation, recruitment strategies, dissemination plans and messages). The group met approximately every 6 months (11 community members) • Trial steering committee (trial oversight). The group met approximately every 6 months. (3 community members, including co-chair) • Trial management group (research design and delivery, analysis of data, writing up and dissemination). The group met approximately every month. (All 11 CEG members invited and provided with minutes) • Independent data monitoring committee (trial oversight). The group met three times. (1 community members)	• Research design and delivery • Ethics • Recruitment • Data collection • Analysis of data (interpretation) • Writing-up • Dissemination
3.	Extended Community Engagement Meeting in June 2013 at the Medical Research Council in London – this involved people working in the media or on dating sites (8 community members)	• Recruitment
4.	Participant involvement meeting: a. 12th November 2013: ‘Future Options for PROUD’ by teleconference and webinar (8 participants) b. 19th March 2014: ‘Future HIV prevention research priorities’ at Terrence Higgins Trust in London (17 participants) c. 9th & 11th September 2014: ‘Study design and data collection tools for a larger PROUD trial’ at the Medical Research Council (in London on 9th and by teleconference on 11th September) (12 participants) d. 16th & 18th February 2015: ‘PROUD Study Results’ at the following locations: i. **Manchester:** 16th February (5 participants) ii. **London:** 16th February (24 participants) iii. **Brighton:** 16th February (14 participants) iv. **Sheffield**: 18th February (4 participants) e. 25th June 2015: ‘World Health Organisation PrEP Implementation Guidelines’ at 56 Dean Street clinic (11 participants) f. February 2017: review of the interpretation of an analysis of self-reported depression during the study (13 participants online)	• Agenda (future research) • Research design (future trial) • Data collection • Analysis of data (interpretation) • Dissemination
5.	Involved in communicating results of the PROUD study, from February 2015 onwards, including: • Getting involved with making the PROUD film documentary (2 community members and 3 participants) • Attending showings and panel discussions of the film (various locations around the UK) • Speaking to the media or providing information to the media (> 5 community members and > 5 participants) • Getting involved in the World AIDS Day twitter event (December 2015) (1 community members and 1 participants) • Speaking to groups as a participant or community representative of PROUD (> 5 community members and > 3 participants) (various locations around the UK) • Getting involved in advocacy as a PROUD participant (for example through United4PrEP) (> 5 community members and > 5 participants)	• Dissemination
6.	Reviewed a new PROUD study questionnaire by email in November/December 2015 (6 community members and 6 participants)	• Research design and delivery

An additional challenge for PROUD, was the involvement of people representing the study population, i.e. HIV negative MSM and trans women who were potentially ‘at risk’ of developing HIV, who are in many ways, atypical of patient involvement in clinical trials. They are not routinely in contact with a treating clinician, and while there are several charities that represent the target populations, they had not previously engaged in clinical research [12, 13]. Therefore PROUD took the decision to aim to involve trial participants. Participants were uniquely placed to represent the wider study population, provide perspectives of individuals who may potentially benefit from the research results, and (because PrEP was not available in the UK at that time) provide unique, first-hand experience of using (or wanting to use) PrEP [10]. PROUD involved participants through a range of approaches, including face-to-face meetings, teleconferences, webinars and email. The participant involvement activities were coordinated by the trial social scientist, with Community Engagement Group members facilitating some of the participant meetings. PROUD also aimed to evaluate participant involvement, to learn lessons and make recommendations for future clinical trials.

## Methods

The Guidance for Reporting Involvement of Patients and the Public (GRIPP2) Long Form checklist for this study can be found in Additional file 1.

### Survey development

One author (MG) developed the initial plan for this evaluation. The MRC CTU’s Patient and Public Involvement Group [14], which includes researchers and patients/community members, reviewed and further developed the plans prior to initiation of the evaluation.

We carried out two structured surveys to evaluate the PPI within PROUD; one of participant and community representatives involved in the PROUD study, and the other of researchers from the PROUD team. Both surveys were developed by two of the team members (MG and BH), drawing on a previous survey undertaken within the Medical Research Council Clinical Trials Unit at University College London (MRC CTU at UCL) [15]. They contained a mixture of open and closed questions designed to capture both quantitative and qualitative information on the process and impact of the involvement. The surveys were implemented using online questionnaires in the Bristol Online Surveys system [16]. Prior to wider distribution, other members of the team (AS and CV) reviewed the surveys.

### Data collection

In April 2017 the first survey was launched. All participants and representatives of community organisations who were actively involved in the PROUD study were contacted via email by the PROUD study PPI coordinator. (PROUD participants who had been involved in PPI activities during the trial and had previously agreed to provide follow-up relating to PPI activities had provided their email address to the PPI coordinator). The email included a link to the anonymous online survey. Three reminders were sent to participants and two reminders were sent to community representatives during April and May 2017.

The second survey was launched in May 2017. All researchers who were involved in PPI activities in the PROUD study were contacted by the MRC CTU at UCL’s PPI consultant via a personalised email that included a link to the online survey. Three reminders were sent in June and July 2017.

While PPI activities do not usually require ethical approval, where participant involvement is used it may require ethics review. PPI including participant involvement in PROUD was approved as part of the REC approval for the trial (REC: 12/LO/1289). This is discussed in our previous publication [10]. The surveys were completed anonymously, and those completing them consented to the results being analysed and published. The UCL research ethics committee exempts ‘Research involving the use of non-sensitive, completely anonymous educational tests, survey and interview procedures when the participants are not defined as “vulnerable” and participation will not induce undue psychological stress or anxiety’ from the requirement to seek ethical approval from the committee. As our study met these criteria, ethical approval from the UCL research ethics committee was not sought.

### Analysis

Two authors (MG and BH) reviewed the results from the surveys. Each independently identified themes from the data for each of the questions, which required open text answers. The two sets of themes were then compared and discussed to agree one set of themes. Responses were then coded to these themes.

## Results

### Survey response

Of 544 participants enrolled in the PROUD trial, 88 (16%) were involved in a PPI activity, with 25 of these (28%) involved in more than one activity. All but one of the involved participants had valid contact details and were invited to participate in the evaluation survey (87/88, 99%), of whom, 46 (52%) completed it (Table 2).

**Table 2:** Participant and community representatives

	Participants	Community representatives
**Number involved in PPI**	88	11
**Number responded**	46 (52%)	8 (73%)
**Age**^a^		
18–29	3 (7%)	0 (0%)
30–39	13 (30%)	1 (13%)
40–49	16 (36%)	4 (50%)
50–59	6 (14%)	2 (25%)
60 & over	6 (14%)	1 (13%)
**Are you based in London (within the M25)?**		
Yes	28 (61%)	6 (75%)
No	18 (39%)	2 (25%)
**Type of activity involved in**^a^		
Membership of Advisory Group	3 (7%)	8 (100%)
Participant involvement meetings	32 (70%)	2 (25%)
Communicating results	16 (35%)	6 (75%)
Email review of documents or findings	18 (39%)	4 (50%)
Extended Community Engagement Group with media organisations	2 (4%)	4 (50%)
**Ever been involved in similar processes before**	4 (9%)	4 (50%)
**Do you think your involvement made a difference to the study?**		
Yes	34 (74%)	8 (100%)
No	12 (26%)	0 (0%)
**Did your involvement have an impact on your as an individual?**		
Yes	33 (72%)	5 (63%)
No	13 (28%)	3 (38%)
**If you represented a community organisation in the PROUD study, did your involvement have an impact on the organisation you represented?**		
Yes	N/A	8 (100%)
No		0 (0%)
**Would you have liked to be more involved?**		
Yes	20 (43%)	3 (38%)
No	24 (52%)	5 (63%)
**In future HIV prevention trials, is there anything we should do differently when we involve people?**		
Yes	18 (39%)	3 (38%)
No	28 (61%)	5 (63%)
**Would you recommend being actively involved in such activities to others?**		
Yes	34 (85%)	8 (100%)
No	3 (8%)	0 (0%)
It depends	3 (8%)	0 (0%)

^a^NB. Adds up to more than 100% as people could be involved in more than one activity type

Eleven community representatives were involved in a PPI activity. All 11 were contacted to participate in the evaluation survey, of whom 8 (73%) responded. One community representative was also a trial participant.

The second survey was sent to ten researchers who were involved in PPI activities, including the chief investigator; the co-chair of the trial steering committee; the chairs of the trial management group, community engagement group and independent data monitoring committee, the PPI coordinator and trial team members. Six (60%) completed the survey (Table 3).

**Table 3:** Researcher responses to categorical questions

	Number (%)
**Respondents**	6 (60%)
**Did you have any experience of PPI in clinical trials before this study?**	
Yes	3 (50%)
No	3 (50%)
**Did PPI bring any benefits to the PROUD study?**	
Yes	6 (100%)
No	0 (0%)
**Were there any challenges with PPI in the PROUD study?**	
Yes	4 (67%)
No	2 (33%)
**Has PPI in the PROUD study influenced your decision to involve participants and/or community members in subsequent research studies?**	
Yes	3 (50%)
No	3 (50%)
**Would you have liked participants and/or community members to be more actively involved in PROUD**	
Yes	3 (50%)
No	3 (50%)
**In future HIV prevention trials, is there anything we should do differently when we involve people?**	
Yes	3 (50%)
No	3 (50%)

The overall response rate across the three groups was 55% (60/109). Overall, participants were younger than community representatives with 37% (17/46) of participants aged below 39 years old compared to 13% (1/8) of community representatives. The majority of participants (61%, 28/46) and community representatives (75%, 6/8) were from Greater London. As expected, participants were predominantly involved in participant involvement meetings and community representatives in management committees (Table 2).

### Motivations for getting involved

Participants’ main reasons to get involved in PPI activities included personal reasons in terms of wanting to learn more about PrEP and the PROUD trial, altruistic reasons in terms of wanting to give something back to the PROUD study, and advocacy reasons in terms of wanting to share information about, and raise the profile of, PrEP. Participant responses relating to these three reasons included:

“To be more involved and gain more information through a shared and safe environment.” (Participant 22553581)

“To show support for the whole concept of the PROUD study” (Participant 22254638)

“I had had such a positive experience of taking PrEP, that I felt I needed to spread the word, as it were, from the view point of someone who was benefiting from being on PrEP” (Participant 22448496).

Some participants chose to get involved with activities that felt particularly relevant or personal to them, such as this participant who reviewed the credibility of our interpretation of study findings showing changing levels of self-reported depression during the study.

“I completed the *[PPI]* survey on depression as I thought it was very relevant to my circumstances” (Participant 22396533)

Only a few participants explicitly stated that they got involved specifically to influence the trial:

“I wanted to engage with the research process in a way which felt meaningful and which could have the potential to influence future trials. This was important for me as someone with learning difficulties as I feel like often times we are not well catered for in healthcare and trial settings.” (Participant 22509803)

Conversely, the main reasons for community representatives to get involved in PPI activities were specifically to influence the design and conduct of the trial and to be well positioned to translate the results into policy, for example:

“Ensure that there was LGBT *[(Lesbian, Gay, Bisexual and Transgender)]* community representation and voice” (Community Representative 22693664)

“I believe that PrEP is a key new prevention tool which can change fundamentally the course of the HIV epidemic. I also believe that for PrEP to be introduced as soon as possible, and for implementation to be successful, community understanding and support are essential” (Community Representative 22827959)

We did not ask researchers for their main reasons for implementing PPI activities as it is MRC CTU at UCL policy that all our clinical studies have PPI activities.

### Experience of involvement

Only four participants (4/46, 9%), four community representatives (4/8, 50%) and three researchers (3/6, 50%) had previous experience of PPI type activities.

Two of the participants stated that their experiences of PPI in PROUD did not compare favourably to other experiences:

“It was very poor compared to other initiatives I have been involved in. I feel like the same req *[sic]* individuals would be consulted time and time again” (Participant 22382194)

Whereas community representatives and researchers with previous experience of PPI all rated their experiences of PPI in PROUD very favourably:

“PROUD felt exemplary for community and patient participation” (Community Representative 22510245)

“I think the range of PPI models in PROUD was far more extensive than I have experienced in other trials conducted in the UK that often rely on single models with patient reps on management groups” (Researcher 24391665)

Overall participants’ experiences of PPI were very positive (described with words like ‘good’, ‘great’ ‘enjoyable’, and ‘pleasant’). When asked how they found the experience, several participants commented about the process of involvement, using words such as ‘accessible’, ‘welcoming’, ‘user friendly’, ‘easy’, ‘non-forceful’, ‘simple’, ‘well managed’, ‘comfortable’, ‘inclusive’, and ‘well organised’. Others commented on learning from the experience, using descriptions such as ‘informative’, ‘interesting’ and ‘educational’. Some commented on finding the experience ‘beneficial’, ‘worthwhile’, ‘useful’ or ‘rewarding’. Others highlighted the emotional impact of involvement, using words such as ‘empowering’ and ‘encouraging’.

Participants described their experiences in the following ways:

“I was always very comfortable during them and felt my small contributions were valued and appreciated and found the researchers to be very respectful of everyone, engaged, and responsive to what they heard.” (Participant 22362641)

“Really rewarding, I was so impressed by the ways in which participants were involved in the ongoing delivery of the trial” (Participant 22510245)

However, the two participants who had not rated their experiences favourably commented on problems, mainly related to barriers to involvement for participants out of London:

“Poor, I had to attend London and the feedback appeared to be dominated by the group of guys who knew each other” (Participant 22660776)

“The experience was poor, if you were out of the London area, no one facilitated being able to attend” (Participant 22382194)

Concerns about timing and follow-up of feedback were raised by two other participants:

“Great, although timings made joining real events difficult” (Participant 22376119)

“It was OK. Nice to meet a few others. Nothing seemed to happen as a result of our feedback though.” (Participant 22662269)

All of the community representatives were broadly positive about their experiences of the involvement activities:

“The high degree of community collaboration was heartening and inspiring. I found a real serious commitment to community engagement from the PROUD team” (Community Representative 22827959)

“The openness of *[the Chief Investigator]* and other researchers to be involved with community organisations was refreshing - and helped me understand the issues better.” (Community Representative 22658334)

“It was very good that the formal structure for the study included community representation at all levels” (Community Representative 22692359)

However, this respondent was critical of reluctance from researchers to take on some suggestions from community partners:

“Sometimes the research group was reluctant to incorporate community advice into the protocol and this made trying to help the study difficult - even if eventually the suggestions we made as a group were included.” (Community Representative 22692359)

We did not ask researchers about their personal experience of the PPI activities as most were not directly involved in the activities.

### Impact of involvement on the trial

74% of participants (34/46) and all 8 community representatives thought that their involvement had made a difference to the trial. However, the distinction between ‘participation’ and ‘involvement’ was not clear-cut for participants and some of their responses referred to the impact of their participation in the study instead of just their involvement in PPI activities. Others referred to feeling as if their own contribution was minimal and therefore did not have an impact, as distinct from considering if the overall PPI activity had an impact on the trial. All researchers thought that PPI was beneficial to the trial.

Not surprisingly, community representatives were more likely to refer to the impact of PPI on the trial design, as they were involved in the design and implementation phase:

“I was involved in a couple of crucial decisions. One specific one was obtaining community buy-in for the immediate/deferred design of the study at a time when some community stakeholders were questioning whether this was ethical.” (Community Representative 22509684)

“The patient information was easier to read (lower reading age, higher readability score)” (Community Representative 22692359)

However, a few participants also commented on the impact of involvement activities on the development of trial questionnaires during the study:

“I think in particular it was good to have participants review study documents to ensure that they are fit for purpose.” (Participant 22509803)

Community representatives also highlighted the impact of PPI on study recruitment:

“Community report publicising the study hopefully helped with enrolment” (Community Representative 22692359)

Both participants and community members particularly highlighted the important role of PPI in interpreting and disseminating the trial results, which showed that PrEP was highly effective at protecting participants from HIV:

“It made a difference to the communication of the study.” (Participant 22921641)

“Community involvement in contributing to the way the results were communicated (formal presentations and publications from the group) turned out to also be very important because of the different interpretations of the data coming from different academic partners. On this, the community input raised important issues that affected the final presentations.” (Community Representative 22692359)

Researchers highlighted the beneficial impact of PPI on the entire trial cycle including the study design, development of study materials, recruitment, trial conduct and results dissemination:

“We might have struggled to obtain approval for the design without evidence of community consultation and endorsement, and there were very direct benefits in terms of recruitment following the June 2013 community engagement meeting. Our materials for recruitment were designed through PPI but the trial was also actively promoted during outreach activities” (Researcher 23669498)

“Recruitment- increasing awareness of PrEP and the study. Disseminating results to participants and the public quickly” (Researcher 24276829)

Participants, community representatives and researchers all noted that the PPI during the trial was of critical importance on the transition from research evidence to advocacy:

“Writing and speaking about PrEP from the point of view of someone who was actually taking PrEP helped to get the message out there. When I first started taking it, a large proportion of the gay world were against it. By the time the PROUD study ended, people were much more positive about it” (Participant 22448496)

“The involvement activities bore fruit in effective political pressure and consistent and powerful media messaging - this was essential especially during the difficult period of legal stand-off between NHS *(National Health Service)* England and the community.” (Community Representative 22827959)

“Most importantly and unusually, the people who were involved in the trial PPI, both from NGOs and participants, have been the very people who have been most visible and proactive in translating the research into practice and advocating for PrEP….. Their involvement in the trial PPI meant that there was not a gap between research and advocacy and there was a shared history and knowledge.” (Researcher 24391665)

The main criticisms about the lack of impact were in relation to not receiving feedback on what changed because of the PPI activities and the activities not including participants across the country:

“No feedback on what changed as a result of our discussions” (Participant 22662269)

“No, because it wasn't representative of the whole country and focussed on the easily managed areas where the majority of participant were (London /Manchester)” (Participant 22382194)

Conversely, a community representative from outside of London noted that a positive impact of PPI on the study was:

“That non-metropolitan views were considered” (Community Representative 22671634)

### Impact of involvement on individuals and organisations

72% (33/46) of participants and 63% (5/8) of community representatives reported that the involvement activities had an impact on them. Again, some participants referred to the impact of their participation more so than the involvement activities. However, for some participants, involvement brought the opportunity to build their understanding about HIV and/or PrEP and to share experience:

“Got a window into what (*sic)* a completely different part of society works, compared to what I was used to in my daily life” (Participant 22819970)

“It was great to see the workings around the trial, both as a participant and as a health promotion worker. It was really useful to gain a better understanding of the trials aims and progress”. (Participant 22510245)

Other participants commented on the extent to which both their participation but also specifically their involvement in PPI increased their desire and confidence to be advocates for PrEP:

“It strengthened my resolve to ensure to the best of my efforts that PrEP is rolled out to those in need. It gave me the opportunity and space to critically assess the arguments for and against and develop an independent opinion sufficiently robust to challenges to assist in the wider cause. I am proud of my involvement and hope that the messages shared through my TV appearances and news articles have affected others and influenced attitudes towards PrEP” (Participant 22552233).

Three participants reported engaging in HIV related voluntary work and other PPI roles as a result of their experiences in the PROUD PPI activities.

Community representatives said that the involvement had provided personal development, personal satisfaction, opened up doors for future involvement, and:

“It reinforced and deepened for me my awareness of the vital importance of community engagement and it also helped get me and my organisation known to members of the communities affected by HIV”. (Community Representative 22827959)

All community representatives reported that the involvement activities had a positive impact on their organisations. The organisational impact related to changing organisational attitudes, an enhanced reputation or an improved relationship with researchers:

“Regular engagement with the trial, and transparent reporting, gave me the up to date information I needed to help change attitudes within my organisation around PrEP.” (Community Representative 22510245)

“I didn't really 'represent' [organisation] but my presence on PROUD secured their reputation as being an authoritative source of information on biomedical prevention” (Community Representative 22509684)

We did not ask researchers about the impact of PPI on them personally, as when we have asked this in previous surveys of researchers they have just repeated what they said when asked about the impact of involvement on research. However, a few researchers did mention personal benefits:

“The PPI in PROUD was a great experience. It is invigorating as a researcher to get constructive input from community members and participants, and I value that personally in terms of having a path through which to hear alternative perspectives” (Researcher 24391665)

Researchers stated that the experience of PPI in PROUD reinforced the benefits of PPI, and half said this would influence them in future studies:

“Seeing the benefits of participant and community engagement for PROUD is encouraging and highlights the potential benefits for other studies” (Researcher 24073844)

“PROUD really highlighted the benefit of including a range of PPI models and this is what I will take forward to subsequent studies” (Researcher 24391665)

### Importance of PPI

45% (21/46) of participants and 38% (3/8) of community representatives said they would have liked to be more involved in PPI activities. The main barriers were location, time, cost, expertise, desire for anonymity (as barrier to media work) and understanding the expectations of involvement. Half of the researchers (3/6) said they would have liked more involvement and this mainly related to including more community organisations:

“Ideally it would have been good to have more representation from smaller NGOs representing particular population groups such as MSM from BAME populations. In truth, with limited funding, we attracted the large, well-funded NGO’s as ‘community’ representatives, and had limited success in attracting the smaller less well funded NGOs” (Researcher 24391665)

79% of participants and all community representatives said that they would recommend being actively involved in such activities to others:

“Surely I would. It's a great thing to do.” (Participant 22665516)

“Yes - a rewarding and challenging opportunity to speak for what you believe in.” (Participant 22552233)

“Yes - a life changing experience in my case!” (Participant 22881322)

“Absolutely yes. Essential. Involvement can enable people to understand the complexities and challenges of clinical research and the amount of work that is involved in getting good evidence that can ultimately help improve healthcare.” (Community Representative 22692359)

### Recommendations for PPI in future trials

Overall, the findings suggest that the level of PPI activities in PROUD were appropriate with half of the researchers and around 60% of the participants and community representatives agreeing that in future HIV prevention trials, there was nothing that we should do differently (Tables 2 and 3). However, approximately 40% of participants and community representatives (22/54) made recommendations for how PPI could be improved in future trials, although only six participant responses related specifically to PPI as opposed to the trial more generally.

Suggestions for improvement from participants included advertising participant involvement earlier such as at enrolment, increasing awareness of the opportunity to get involved, and advanced notice of meetings. The need for more opportunities for involvement outside of London, were echoed by participants and researchers:

“While we offered a range of ways in which participants could get involved in PPI, the most fruitful model was through face to face participant involvement meetings. All but one of the participant involvement meetings were exclusively held in London… Ideally it would have been preferable to arrange more regional participant involvement meetings. This would have been possible via our NGO partners if we had resources to support it.” (Researcher 24391665)

Suggestions for improvement from community representatives included involving community groups more in the protocol development process, including potential ‘users’ (such as participants) on formal advisory committees, and one recommendation to give more weight to suggestions that come from the community, than from researchers:

“Perhaps have a lower threshold to community suggestions if these are different to the research group consensus - they come from a different experience base and it is hard work trying to explain to a group of experts why there might be other options”(Community Representative 22692359)

We specifically asked participants and community representatives about the management of confidentiality of PPI representatives, but most either did not have an opinion or felt this was sufficiently handled in the trial. Some participants and community representatives made suggestions about offering more opportunities to act as anonymous spokespeople and to develop agreements upfront.

Half of the researchers (3/6) noted specific challenges in conducting PPI and made recommendations for future trials. These mainly related to the need for more funding for PPI; the inclusion of participant representatives on advisory boards; structured evaluation of the impact of PPI during the trial; the need for more engagement in PPI by all researchers; the need for PPI expertise to facilitate involvement activities and the possibility to offer training and mentoring in PPI. One researcher also noted the need for better engagement with organisations that reflect the entire participant population and beyond:

“I think we did a very good job at including a range of representative voices of gay and other MSM in PROUD. However, we did not do enough to include the voices of trans women or to make the trial clearly trans inclusive. I think largely due to this we only enrolled 3 trans women and this was a missed opportunity… given PrEP is effective for all groups, and the trial results have implications for PrEP access for everyone at risk of HIV in the UK, we should have considered including NGOs representing the needs of heterosexual women and men at some point during the trial” (Researcher 24391665)

Overall, participants, community representatives and researchers alike, described PPI in the PROUD trial as effective and impactful:

“Proud to have been involved in PROUD” (Community Representative 22208812)

## Discussion

### Summary

We have demonstrated that in the PROUD study, a multiple-model approach to PPI, using minimal resources, brought substantial benefits. In particular, the involvement of both participants and community representatives was essential to ensure that both younger HIV-negative voices (participants) and experienced voices (community) with understanding of clinical trials and of PPI were heard. This enabled involvement in PROUD to impact across the study, at all stages, and on policy-makers. Importantly, involvement also had a positive impact on the individuals and organisations concerned.

### Context

Evidence of the impact of involvement in health research has begun to emerge in the literature [9, 15, 17–20]. Positive impacts include a sense of improved quality, appropriateness, and relevance of research; improving the research question, study design, participant information, recruitment strategies, and interpretation and communication of the results. They also noted that the trials were more relevant to the potential beneficiaries of the research. They have also identified challenges posed by PPI – for example power-struggles and conflict in decision-making, managing confidentiality, prioritising of opinions, and resources. However, very little has been reported regarding the impact of involvement of trial participants in PPI [10].

The involvement of both participants and community representatives in PPI is seemingly uncommon in the UK. The current INVOLVE guidance says involving participants is usually not appropriate due to concerns that it will “compromise both the researcher and the person involved” [21], although our experience in PROUD has led us to call for INVOLVE to reconsider this advice [10]. PROUD is the first example of such involvement in a UK clinical trial that we are aware of, however in sub-Saharan Africa, particularly in relation to HIV clinical trials, such an approach is more commonplace. Indeed it is recognised and encouraged in GPP guidelines for biomedical HIV prevention trials, tuberculosis (TB) drug trials, TB vaccine research, and trials of emerging pathogens [2, 22–24]. Perhaps in the UK we have been slow to adopt similar strategies for PPI, however, we believe there is much to gain, in particular where the voices we wish to hear and the communities we seek to involve have been traditionally excluded from PPI.

### Strengths

In total, 60 participants and community representatives and researchers participated in this evaluation of the PPI that took place in PROUD. As evaluation of involvement in clinical trials is rare in the literature, we believe this is one of the largest scale evaluations to be reported. Uniquely, our involvement and the evaluation of it has involved participants, and covered a trial using multiple models of PPI. The scale therefore has enabled us to evaluate multiple perspectives from all stakeholders and to draw out similarities and differences between the respondents. Our survey design, with the use of free text responses, facilitated this and we have conducted both qualitative and quantitative analysis of the data gathered. We believe this is the first such evaluation conducted for a UK clinical trial.

### Limitations

Despite the number of responses included in this evaluation, the delay between completing the trial (October 2016) and carrying out this evaluation (April – July 2017) inevitably contribute to the response rate (52% of participants; 60% researchers and 73% community) being lower than we may have liked, particularly for the participants. The delay possibly also contributed to some confusion seen in the responses of some PROUD participants between evaluation of their experiences of being a participant in the trial and evaluation of their active involvement in the PPI. While this has meant some of the data has been more difficult to use, overall, the reported experiences of involvement have still been very positive.

### Future steps

This evaluation has highlighted lessons for our future studies in terms of both how PPI is managed and evaluated. Our results show that commitment to PPI from senior level researchers, or clinicians along with an expert to facilitate PPI activities were all widely appreciated and so we will endeavour to ensure this in all future trials. However, we should aim to diversify involvement in future trials – by hosting meetings or events in varying locations and by seeking to engage with smaller organisations and NGOs. This should open up access to those less able, or willing, to travel to London, and ensure that regional variation in opinion or experience is heard. Resources will be needed to support this and should be included in study budgets. It would also be useful to monitor resources spent on PPI in future studies to help identify cost-effective approaches. We also note that whilst this evaluation has been valuable, we should seek to rigorously evaluate the impact of PPI in real time. This would enable us to act upon feedback whilst the study is ongoing and may potentially have reduced or avoided some of the more negative findings and opinions expressed in this evaluation. A real time evaluation could also capture the variance of opinions on given topics and document when and why community suggestions are not taken forward. Each of these additional approaches would however require additional funding and prospective planning of PPI activity. Another consideration for future studies should also be the inclusion of community members with experience of PPI on grant applications to support PPI activities throughout the lifecycle of trials. This is being increasingly requested by funding bodies.

## Conclusion

The responses to our surveys on the PPI activities within the PROUD trial are overwhelmingly positive, with the majority of participant and community representatives and half of the researchers agreeing that in future HIV prevention trials no change should be made to how we involve people. The findings demonstrate the benefits of involving clinical trial participants and wider community representatives as active partners in PPI. Such involvement is already happening in trials in Africa; however, PROUD is one of the first United Kingdom-based clinical trials to adopt this approach. In particular, our results support this approach to PPI in other trials that recruit from populations that researchers are less experienced in engaging with, or those testing a novel treatment or approach not available outside the trial. Our results also highlight the need for good planning and resourcing of PPI in trials – in particular, where multiple models of involvement are used. Researchers should also make prospective plans to evaluate and adapt the PPI arrangements within their trials and appropriately resource these.

## Supplementary information

**Additional file 1.** GRIPP2 Long form checklist.

## Data Availability

The MRC Clinical Trials Unit at UCL is committed to sharing clinical study data for additional, ethical research with justified scientific objectives. We have a controlled access approach whereby researchers make formal applications for data sharing. More details on the process can be found on the MRC CTU website [25].

## Abbreviations

BAME: Black Asian and Minority Ethnic
GPP: Good Participatory Practice
GRIPP2: Guidance for Reporting Involvement of Patients and the Public
HIV: Human Immunodeficiency Virus
INVOLVE: INVOLVE is a national advisory group that supports greater public involvement in National Health Service, public health and social care research
LGBT: Lesbian, Gay, Bisexual and Transgender
MRC CTU at UCL: Medical Research Council Clinical Trials Unit at University College London
MSM: Men who have sex with men
NGO: Non-governmental organisation
NHS: National Health Service
PPI: Patient and Public Involvement
PrEP: Pre-exposure prophylaxis
PROUD: PRe-exposure Option for reducing HIV in the UK: an open-label randomisation to immediate or Deferred daily Truvada for HIV negative gay men
REC: Research Ethics Committee
TV: Television
UK: United Kingdom
UNAIDS: Joint United Nations Programme on HIV/AIDS
